# Addressing policy barriers to scaling up needle and syringe programmes: a global call to action

**DOI:** 10.1016/S2214-109X(25)00433-4

**Published:** 2025-12-09

**Authors:** Guillaume Fontaine, Emma Day, Niklas Luhmann, Annie Madden, Keith Sabin, Andrew Scheibe, Mark Stoove, Peter Vickerman, Ernst Wisse, Jason Grebely, Natalie Taylor, International Network on Health and Hepatitis in Substance Users Policy Day Working Group, Philip Bruggmann, Judy Chang, Emily Christie, Monica Ciupagea, Colleen Daniels, Emma Day, Jason Grebely, Kim Green, Kiera Gustafson, Jennifer Hasselgard Rowe, Niklas Luhmann, Annie Madden, Natasha Martin, Susie McLean, Keith Sabin, Andrew Scheibe, Heather Marie Schmidt, Mat Southwell, Mark Stoove, Karin Timmermans, Anna Tomasi, Beatrix Vas, Annette Verster, Peter Vickerman, Ancella Voets, Ernst Wisse

**Affiliations:** a McGill University, Montréal, QC, Canada; b Lady Davis Institute for Medical Research, Montréal, QC, Canada; c Ottawa Hospital Research Institute, Ottawa, ON, Canada; d Kirby Institute, University of New South Wales, Kensington, NSW, Australia; e International Network on Health and Hepatitis in Substance Users, Barangaroo, NSW, Australia; f WHO, Geneva, Switzerland; g International Network of People Who Use Drugs, Barton, ACT, Australia; h UNAIDS, Geneva, Switzerland; i TB HIV Care, Cape Town, South Africa; j University of Pretoria, Hatfield, South Africa; k University of Bristol, Bristol, UK; l Burnet Institute, Melbourne, VIC, Australia; m Médecins du Monde, Paris, France; n Implementation to Impact (i2i), School of Population Health, University of New South Wales, Kensington, NSW, Australia

## Abstract

Needle and syringe programmes (NSPs) are effective, affordable solutions for preventing the transmission of blood-borne viruses among people who inject drugs. Yet, global NSP coverage remains extremely low; only 2% of people who inject drugs live in countries with high coverage, and many low-income and middle-income countries do not have NSPs. This Health Policy reports outputs from an international working group who used implementation science approaches to prioritise barriers and co-design solutions to scale up NSPs across three domains: global policy, national policy, and procurement. We present six barriers and 11 strategies that align commodity selection and procurement with the needs and preferences of people who inject drugs, strengthen national commitment and regulatory environments, and improve forecasting and market access for preferred products. We provide sector-specific actions for funders, governments, procurement agencies, implementers, community networks, and researchers. Scaling up NSPs is essential for achieving global infectious disease-elimination goals and improving health outcomes among people who inject drugs.

## Introduction

Globally, an estimated 14·8 million people had recently injected drugs in 2021,^1^ placing them at high risk of HIV and hepatitis C virus infections, and substantial morbidity and mortality.^2^, ^3^, ^4^ Needle and syringe programmes (NSPs) are effective and cost-effective for HIV and hepatitis C prevention, particularly when combined with opioid agonist therapy.^5^, ^6^, ^7^, ^8^, ^9^ Yet, in 2022, only 2% of people who injected drugs lived in countries with high NSP coverage (defined as >200 sterile needles and syringes per person who injects drugs per year), and 62% of low-income and middle-income countries did not have an NSP.^10^, ^11^

NSP policies frequently fail to meet demand due to inaccurate quantification, misalignment with community preferences, restrictive distribution and return policies, inadequate funding, and punitive regulations,^12^, ^13^ contributing to unsafe injecting and undermining efforts to eliminate HIV and hepatitis C virus.^12^, ^13^ Criminalisation of drug use, stigma, harmful law enforcement practices, little political will, and insufficient funding further impede scale-up.^12^, ^13^, ^14^, ^15^, ^16^ Even in countries with established NSPs, restrictive policies, such as low syringe distribution quotas and reluctance to support secondary peer–peer distribution, reduce their impact.^12^, ^13^, ^17^

Instability of key global funding streams (eg, the US President's Emergency Plan for AIDS Relief [PEPFAR]) and constrained capacity at some multilateral agencies, alongside reductions in development assistance in several countries, have disrupted harm-reduction services, particularly in low-income and middle-income countries that were receiving development aid.^18^, ^19^, ^20^, ^21^ The impacts of these funding cuts have been modelled to have major negative impacts on a range of health outcomes globally.^22^ In the absence of international donor funding, NSPs are often deprioritised for domestic financial support, especially during periods of fiscal austerity, despite being a cost-effective and life-saving public health intervention. Political factors and strict law-enforcement measures can curtail NSPs, deter service use, and exacerbate health risks, reinforcing stigma and inequities.^15^, ^23^, ^24^

To address persistent and systemic barriers to the scale-up of NSPs globally, the International Network on Health and Hepatitis in Substance Users (INHSU) convened a multidisciplinary working group. This group aimed to catalyse collaboration among multilateral agencies, funders, implementers, researchers, and community-led networks to identify contextual barriers to high-coverage NSPs and co-design solutions aligned with the values and preferences of people who inject drugs. This work targets six audiences: global funders and multilateral agencies, national governments, national procurement and supply chain agencies, programme implementers, community-led networks, and researchers and clinical partners. The remit of the group was to focus on access to sterile injecting equipment within NSPs. Many NSPs also provide overdose prevention, wound care, and safer smoking supplies; the financing and procurement principles we propose are transferable to those services. This Health Policy aims to prompt global organisations to revise policy and funding frameworks to reflect the needs and preferences of people who inject drugs; support governments to address capacity gaps and use data-driven tools to forecast commodity needs; and improve procurement to ensure acceptable, equitable, and reliable commodity supply.

## Methods

### Multisectoral engagement

26 members were recruited to the INHSU Policy Day Working Group through purposive, snowball-augmented sampling. Inclusion criteria covered experience in policy and financing decision making, programme implementation and service delivery, procurement and supply chain management, legal or policy reform, and lived or living experience with injecting drugs and leadership in community-led organisations. We prospectively targeted balance across roles and geography. Invitations were issued via INHSU lists, regional networks, and direct referrals; sampling closed once role and regional balance targets were met. The resulting group brought together representatives from multilateral and global health organisations (eg, WHO, UN Office on Drugs and Crime, UNAIDS, and The Global Fund to Fight AIDS, Tuberculosis and Malaria), academic and research institutions, civil society organisations, and peer-led or community-led networks of people who use drugs. The full working group membership is presented in the appendix (p 2). Their contributions began in March, 2024, with a combination of online synchronous and asynchronous activities, including a virtual workshop held on Sept 2, 2024, and a series of one-on-one informal, virtual consultations conducted throughout the year. Building on this foundation, INHSU hosted a policy day workshop on Oct 7, 2024, in Athens, Greece. The in-person event convened 54 participants. Not all members of the 26-person working group attended; individual attendance is indicated in the appendix (p 2). Recruitment for the workshop intentionally broadened representation through an open call via INHSU channels and targeted invitations to fill role and regional gaps, prioritising community-led and peer-led networks of people who use drugs and programme or technical advisors. A summary of participant characteristics is presented in the appendix (p 3). Participants came from across all six WHO regions and included a mix of high-income, middle-income, and low-income settings. Nearly one-third of attendees were from peer-led or community-led networks; additional participants included researchers, programme implementers, multilateral agency staff, and government officials.

### Consensus-building method

We conducted a two-stage consensus-building process informed by implementation science frameworks and guided by the modified nominal group technique (mNGT)^25^, ^26^ to prioritise barriers and co-design actionable strategies for the scale-up of high-coverage NSPs. Our method aimed to preserve the mNGT's defining features (ie, structured process and independent quantitative prioritisation), while introducing modifications (ie, asynchronous idea capture, multicriteria scoring, virtual polling, integration with implementation-science frameworks, and an action-planning phase) necessary for a geographically dispersed, policy-focused audience.^27^ An a priori protocol was lodged with the INHSU Secretariat on July 9, 2024, building on implementation-science workshops led by the facilitator team.^28^, ^29^ The appendix (p 4) provides an overview of the method.

### Guiding frameworks

Implementation science provides a structured and evidence-informed approach to overcoming barriers in health policy and service delivery.^30^, ^31^, ^32^, ^33^ We used the consolidated framework for implementation research (CFIR) to categorise barriers and facilitators to NSP scale-up across five domains: intervention characteristics, outer setting, inner setting, individual characteristics, and implementation processes.^34^, ^35^ The expert recommendations for implementing change (ERIC) taxonomy, which organises 73 discrete strategies (eg, build a coalition and conduct outreach) into thematic clusters, offered a common language for strategy specification.^36^ ERIC allows researchers, implementers, and decision makers to specify exactly which strategies should be used to promote uptake and sustainment, compare strategies across contexts, and replicate effective strategies in new settings.^36^ To align barriers and strategies, we applied the CFIR–ERIC matching tool, which assigns endorsement scores based on expert consensus (eg, matching an external policy barrier with the strategy of building a coalition yields a 33% endorsement rate, which, although modest, is the third highest ranked of 73 strategies).^37^ Finally, Proctor's framework guided the specification of selected strategies to support the scale-up of NSPs across different domains, clarifying actor, action, target, timing, and intended outcomes.^38^

### Procedures

Barriers to scaling up NSPs were first identified through feedback from members of the INHSU Policy Day Working Group, followed by asynchronous clarifications in a shared document. Barriers were then collated and CFIR-coded by the facilitation team (GF, ED, and NT) and the full list was circulated to group members. During a virtual workshop held on Sept 2, 2024, we conducted a live discussion to identify any ambiguities for barriers and make refinements. Working group members then used a private, structured, multicriteria rating system in a purpose-built online survey to prioritise barriers to scaling up NSPs. Each barrier was assessed on a 5-point Likert scale across four dimensions: severity, geographical or demographic reach (widespreadness), feasibility (effort to address), and potential for positive knock-on effects. Additionally, participants described outcomes by which the programme could be deemed successful for each barrier group to identify outcomes. A composite score was calculated to prioritise barriers. We computed a weighted mean: 0·50 × feasibility + 0·30 × severity + 0·10 × reach + 0·10 × positive knock-on effects. All 15 barriers were then ranked by this score, and the top two per domain were advanced to strategy development and policy action.^28^, ^29^

The top six barriers were brought forward to the in-person INHSU policy day. 54 participants engaged in six facilitated roundtable discussions (nine participants per table) over the course of a structured, 4 h session. The workshop was organised into three key steps. First, during the idea-generation stage, participants discussed two prioritised barriers per table and proposed potential solutions without initial concern for feasibility. Second, during strategy selection and refinement, participants used a predeveloped working document, prepared by GF, ED, JG, and NT, to review three to five premapped strategies for each barrier. They narrowed down to one to two preferred strategies per barrier based on relevance and perceived utility. Finally, during implementation planning, participants defined how each selected strategy would be operationalised in practice, specifying roles and responsibilities, required resources, and feasible timeframes, with a strong focus on actionability. For example, the barrier of “lack of political will from national governments to allocate or request resources for NSP implementation or scale-up” was mapped to the CFIR construct “external policy and incentives” (outer setting; appendix p 10). Different strategies were proposed based on the CFIR–ERIC matching (eg, build a coalition of interest-holders such as non-government organisations, local governments, and peer-led or community-led networks of people who use drugs to promote investment in harm reduction and facilitate evidence-informed consensus-building discussions between governments, health providers, and peer-led or community-led networks of people who use drugs to align on the importance of NSPs). The feasibility of each strategy was assessed against its alignment with financial, human, and material resources, potential support from key interest-holders and policy makers, implementation timelines, interest-holder capacity and training, and overall complexity. These considerations guided structured discussions during the in-person workshop.

To further refine strategies after the workshop, additional one-on-one consultations with working group members representing community-based organisations, researchers, programme implementers, and multilateral agencies were conducted by ED over a 4-month period. Final strategy specification was conducted following recommendations for specifying and reporting implementation strategies.^38^ Examples to illustrate key strategies (panel) were derived from group discussions and one-on-one consultations.

Panel **Illustrative models and case examples for key strategies (S)**

**Global policy and guidance**

*UN resolution on harm reduction (S3)*

Recent recognition (Commission on Narcotic Drugs resolution 67/4) of evidence-based harm reduction measures strengthens the basis for standalone budget lines and permissive access rules in donor and domestic financing frameworks.^39^

*Strengthen knowledge for funders (S4)*

Evidence syntheses and guidance framing for people who inject drugs from the European Centre for Disease Prevention and Control (ECDC) and the European Monitoring Centre for Drugs and Drug Addiction (EMCDDA; 2023 update)^11^ and the UNAIDS thematic brief^40^ support policy revision and dedicated funding lines.

**Permissive access frameworks and service models**

*Policy levers and diversified delivery (S7)*

European guidance (ECDC and EMCDDA) supports pharmacy, alternative outlets, and other low-threshold modalities for high-coverage programme provision.^11^

*Example model: Australia (S7)*

Dispensing machines providing sterile equipment anonymously provide out-of-hours and stigma-reducing access.^41^

*Example model: Ireland (S7)*

National pharmacy needle-exchange network shows rapid scaling through existing outlets.^42^

**Procurement and quantification**

*Quantification methods and coverage targets (S8)*

The UN Office on Drugs and Crime/WHO/UNAIDS Technical Guide details coverage calculations (“number of needles and syringes distributed per person who injects drugs per year”) and data sources for quantifying needs and setting realistic annual targets.^43^ WHO set targets for the number of needles or syringes distributed per person who injects drugs, per year, at 200 by 2020 and 300 by 2030, as part of a comprehensive harm reduction programme.^44^

**Community-preferred needle and syringe programme commodities**

*National guidance: Canada (S10, S11)*

Specifies low-dead-space (minimal space between the needle and the plunger when fully depressed) options, appropriate gauge and length mix, and complete preparation and disposal kits; endorses distribution of a comprehensive suite of commodities.^45^, ^46^

*National guidance: UK (S10, S11)*

Recommends a full range of sterile equipment with low-threshold access and provides a template for tender specifications and service delivery.^47^

*Values and preferences work (S11)*

Unitaid's standardised tools capture community preferences (eg, low-dead-space options, gauge and length mix, preparation and disposal kits, low-threshold delivery), yielding procurement-ready product profiles for tenders and service design.^48^, ^49^, ^50^

## Results

### Key multilevel barriers to scaling up NSPs

15 barriers to scaling up NSPs were identified across global policy (n=5), national policy (n=6) and procurement (n=4). The list of these barriers is provided in the appendix (p 5). Six high-priority barriers were taken forward for strategy development (two per domain; figure).

**Figure:**
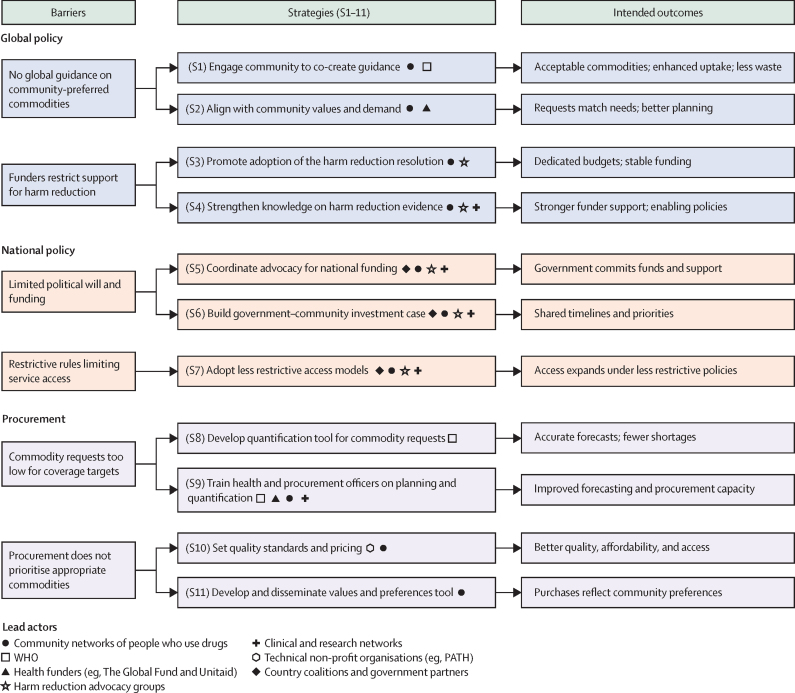
Barriers, strategies, and intended outcomes for scaling up NSP procurement and implementation Key barriers at the global policy, national policy, and procurement levels to 11 strategies and their intended outcomes are mapped. Barriers (left column) describe systemic challenges that limit access to NSPs. Strategies (middle column) are numbered S1–11, expressed in action-oriented terms, and linked directly to one or two barriers. Symbols next to each strategy identify the lead actor groups responsible for implementation. The right column presents the intended outcomes of each strategy, streamlined to highlight both immediate and long-term effects (eg, improved acceptability, stability of funding, or expansion of access). More detail on each strategy and expanded descriptions of outputs and outcomes are provided in the appendix (pp 6–8). NSP=needle and syringe programme.

At the global level, two barriers dominate. First, there is no global guidance on selecting community-preferred NSP commodities. By preferred commodities, we mean a locally tailored mix of fixed-needle and detachable syringes, appropriate gauges and lengths, low-dead-space options (designed to have minimal space between the needle and the plunger when fully depressed), and preparation and disposal kits, packaged discreetly and in sufficient quantities to support secondary peer distribution.^11^, ^45^, ^46^, ^47^, ^51^ In the absence of such guidance, countries often default to the cheapest or most familiar items, resulting in narrow catalogues (eg, heavy ordering of 1 mL fixed-needle syringes) that compromise vein health, safety, and uptake.^51^ Second, restrictive global funder policies still limit or discourage direct support for NSPs and harm reduction, particularly in the context of low-income and middle-income countries receiving global funding. Where NSPs are folded into HIV prevention budget lines without a distinct coverage indicator or budget, applicants struggle to justify required commodities, peer-led engagement, or alternative delivery models, weakening accountability for coverage.

At the national level, the first barrier prioritised is limited political will, reflected in the absence of ring-fenced budgets or donor requests for NSPs, which constrains implementation and scale-up nationally. The second barrier concerns restrictive access frameworks and service models that depress coverage. Examples include identification mandates that exclude people without documents, 1:1 exchange rules that cap commodities below need and discourage returns, and visible policing near services that deters attendance.

For procurement, two barriers were prioritised. First, the quantities of NSP commodities requested by countries from global funding organisations are often insufficient to meet WHO coverage goals. Second, the procurement processes at the country level rarely prioritise community-preferred and appropriate commodities. These barriers, linked to weak quantification methods and short planning horizons, produce stock-outs or rationing, which in turn sustain reuse and sharing and undermine safe coverage levels.

Although our focus was policy and procurement, participants also noted substantial organisational-level challenges. These challenges included constrained operating hours, poor service location placement, confidentiality concerns, stigma, disparities in urban versus rural access, rarity of gender-responsive and differentiated service models, and scarce co-design with people who inject drugs. Addressing these barriers is crucial to ensure high coverage by making NSPs accessible, equitable, and responsive to the needs of people who inject drugs.

## Strategies to scale up NSPs

To address the priority barriers, we developed 11 strategies spanning global policy, national policy, and procurement (figure; additional details in the appendix [pp 6–8]). Together, these strategies provide a framework for addressing some of the most pressing challenges, ensuring that NSPs are appropriately resourced, aligned with demand, and effective in meeting harm-reduction goals.

### Global policy strategies

The four global policy strategies developed focus on addressing the absence of community-preferred NSP commodity selection and procurement guidelines and restrictive policies limiting NSP funding (figure).

First, there is an urgent need to engage people who inject drugs in drafting global NSP commodity selection and procurement guidelines to ensure that commodities selected align with community needs and preferences. Enacting this strategy could build trust between funders, governments, and communities and foster more inclusive and effective harm reduction policies. A related strategy is strengthening The Global Fund's NSP funding-request process to require country applications to justify NSP commodity types and quantities with community preference data and demand estimates, rather than relying on default catalogues or historical volumes. Unitaid's values and preferences work^48^, ^49^, ^50^ provides a useful global reference. These data could be distilled into pragmatic tools to guide local consultative processes that ensure consistency and meaningful engagement, ideally coordinated by a community-led organisation. The intended result is a better-fit product mix, higher uptake, and less wastage, with more accurate allocations and stronger planning capacity compared with current NSPs.

In parallel, advocacy efforts must push for global funding bodies, such as The Global Fund and PEPFAR, to formally recognise harm reduction as a standalone funding priority, independent of HIV funding programmes. This shift would provide dedicated, stable funding for NSPs, preventing fluctuations tied to changing HIV priorities. At the same time, noting the policy day predated recent funding changes in relation to PEPFAR, greater attention to domestic budgeting and sourcing of harm-reduction technologies might also be required. In the context of growing global uncertainty ranging from political instability to shifting donor priorities, governments must step up and take greater responsibility for funding and sustaining harm-reduction services within their own borders. Additionally, improving global funders’ understanding of the benefits of high-coverage NSPs is essential to sustaining investment. Direct engagement through educational sessions and global conferences can strengthen advocacy, allowing funders to hear from experts and leaders from the people who inject drugs community about the real-world impact of harm reduction.

### National policy strategies

The three national policy strategies developed focus on increasing political will, removing restrictive policies and practices, and enhancing NSP accessibility (figure).

A key strategy is advocating for governments to allocate and request dedicated resources for NSP implementation and scale-up. Building a coordinated, multistakeholder coalition would strengthen the credibility and reach of advocacy efforts, ensuring consistent messaging and alignment across sectors. Developing evidence-based materials tailored with country-specific epidemiological and cost data (eg, local HIV and hepatitis C incidence and projected savings from reduced hospitalisations) would further enhance engagement with policy makers. Striking a balance between global best practices and national priorities is essential to make these efforts impactful and politically feasible. Direct engagement of senior officials in health, social, development, and criminal justice services through high-level policy dialogues or parliamentary briefings to present the investment case for harm reduction and emphasise the cost-effectiveness of NSPs is equally crucial. These dialogues should be supported by short, visually compelling briefs that summarise local data and illustrate concrete funding gaps. Inviting multilateral organisations to participate in these discussions can add technical credibility and aligning national policies with global harm reduction strategies will facilitate stronger commitments than are currently common from both governments and funders.

National community-led networks of people who use drugs are important partners in shaping NSP policies and implementation strategies. Supporting and meaningfully involving these networks in policy design, implementation, and monitoring processes is essential. This engagement could be accomplished via dedicated funding lines, representation on national technical working groups, and capacity-building grants. Restrictive policies such as 1:1 syringe exchange requirements and identification mandates must be revised to remove unnecessary barriers to NSP access. Jurisdictions that have authorised secondary peer distribution, pharmacy-based provision, or vending or dispensing models report greater reach and improved continuity of supply, underscoring the value of flexible access pathways. Coordinated advocacy and education efforts involving community networks can support governments to update national guidelines to reflect international best practices.

### Procurement strategies

The four procurement strategies developed focus on ensuring the adequate supply of appropriate NSP commodities, improving procurement processes, and strengthening national capacity (figure).

A key priority is ensuring that commodity selection processes are grounded in evidence and community values, rather than availability or cost alone. The development of robust quantification tools can help align commodity requests with actual demand, optimising resource allocation and minimising supply gaps. Integrating these tools, alongside pragmatic values and preferences assessment tools, into WHO's forthcoming NSP technical guide and The Global Fund application process would encourage adoption of evidence-based approaches to commodity requests. Donors and governments should ring-fence resources and provide technical assistance so that NSPs can run regular preference check-ins and aggregate results for planning. Strengthening procurement capacity is also essential. Training national health and procurement officers on commodity planning, strategic sourcing, and distribution would help align national procurement systems with global best practices and support sustainable programming. WHO is well positioned to lead these efforts.

Additionally, improving market access to affordable, high-quality syringes and needles is crucial. Establishing global quality standards, pricing benchmarks, and negotiated access terms would enhance supply chain stability and promote equitable access. Supporting local manufacturing could further reduce reliance on imports, increasing supply security. Finally, additional capacity-building efforts, including more donor support from The Global Fund and others to establish new and sustain existing peer-based networks of people who use drugs (or minimally, funding The Global Fund applicants to meaningfully engage with such communities), is required to develop and deploy community-led values and preferences tools to ensure procurement decisions reflect the needs of people who inject drugs.

## Recommendations and next steps

To translate consensus into implementation, we describe actions for key stakeholders mapped to the 11 strategies (S1–11 in figure) previously outlined. The emphasis is on actions that can be initiated and substantially progressed within 12 months.

For major funders (eg, The Global Fund and Unitaid), harm reduction should be recognised as a distinct budget line in funding requests and grant performance frameworks (S2 and S3, supported by S8). Funding requests should require justification of commodity type and volume based on community values and preferences, and the technical assistance to understand these preferences (eg, microgrants, helpdesk, and tools) should be financed (S2, S11). Funders should also underwrite the development and roll-out of the WHO quantification tool and associated national training, and the hosting of regional helpdesks (S8 and S9).

National governments and policy makers should introduce a ring-fenced NSP budget line in the next fiscal cycle and endorse the benchmark of at least 200 needles per person who injects drugs per year, targeting 300 by 2030 (S5 and S6, supported by S8). Restrictive rules about service access should be removed, specifically 1:1 exchange and identification mandates, and secondary distribution, vending or dispensing machines, and pharmacy-based NSPs authorised (S7). Use of the WHO quantification tool should be mandated for national planning and forthcoming The Global Fund applications (S8 and S9).

National procurement and supply-chain agencies should embed preferred product specifications in tenders (eg, low-dead-space syringes or needles, an appropriate gauge and length mix, and preparation or filtration and disposal supplies) alongside benchmark pricing and execute multiyear framework agreements to stabilise supply (S10). An annual procurement-mix review, combining consumption and preference data, should inform orders and buffer-stock policies to avoid stock-outs (S8 and S11).

Programme implementers (governmental and not-for-profit) should institute a brief, standardised quarterly preference check-in at distribution points, using donor-funded templates and helpdesk support to report aggregated findings upstream (S11, supported by S8 for forecasting). Where lawful, services should expand flexible delivery modalities including peer delivery, vending, dispensing, and pharmacy NSPs and routinely document reach and stock-outs to guide resupply (S7 and S8).

Community-led networks should be resourced to co-lead guideline drafting and product selection and to participate (with compensation) in national NSP advisory groups (S1 and S11). They should lead local preference assessments and validate procurement plans before submission to funders (S11).

Researchers, clinicians, and data partners should deliver rapid evaluations and local investment cases, such as cost per infection averted and budget-impact analyses, built on outputs from the WHO quantification tool (S8 and S9). They should also develop and disseminate concise case studies of policy and procurement reform via key channels to accelerate learning across settings (S1, S4, S7, S10, and S11). Further implementation research efforts are needed to generate high-quality evidence on the local barriers and facilitators and strategies for scaling up and sustaining NSPs, particularly in low-income and middle-income countries in which data are scarce. Additionally, research should inform best practices in NSP procurement, distribution, and engagement with people who inject drugs and identify strategies to engage and sensitise host communities whose resistance might pose a barrier to implementation. These research directions address organisational barriers emerging from the previously held discussions and barriers listed in the appendix (p 5) that were not among the six prioritised targets but remain crucial to sustainable scale-up. Strengthening collaborations between researchers, policy makers, and people who inject drugs community advocates will be essential to ensuring that implementation efforts are informed by lived and living experience, scientific evidence, and real-world challenges.

## Limitations

This Health Policy synthesises priority barriers and strategies to scale up NSPs globally, but several limitations must be noted. First, the prioritisation process focused on six of 15 identified barriers, selected through a structured consensus-building process weighing feasibility, severity, reach, and knock-on effects. This selection does not imply that the remaining barriers such as criminalisation, punitive policing, societal opposition, and absence of differentiated care are less important. Progress on the prioritised barriers might be constrained or rendered ineffective without concurrent attention to these underlying structural determinants. Second, although we incorporated diverse input, including strong representation from community-led organisations, the final prioritisation reflects convergence rather than full consensus. Not all participants endorsed every barrier or strategy, and the synthesis should not be interpreted as a uniform position. Third, the initiative occurred during relative stability in global funding. Since then, the US withdrawal from WHO and cuts to PEPFAR, the US Agency for International Development, and The Global Fund, alongside broader reductions in aid, have created uncertainty for harm reduction financing. These shifts increase risks for NSP sustainability, especially in low-income and middle-income countries historically reliant on donor support. Although the strategies remain valid, their implementation now requires greater emphasis on domestic financing, political commitment, and context-specific delivery models.

## Conclusion

Ensuring that all people who inject drugs have access to sterile equipment for every injection is a proven, life-saving harm reduction strategy, yet it remains critically underimplemented globally. Despite substantial scientific evidence, NSP scale-up continues to be hindered by restrictive global, regional, and country policies, entrenched stigma and discrimination, and chronic underfunding. The consequences of inaction are devastating: new blood-borne virus infections, outbreaks and uncontained epidemics, preventable deaths, and widening health inequities.

We cannot afford to delay; it is time to move from evidence to impact. Targeted policy reforms, sustained investments, tailored implementation efforts, and bold, community-led advocacy are essential to dismantling the barriers that obstruct access to NSPs. Implementation science offers a pathway to strengthen these efforts by identifying what works, for whom, and under what conditions, and by enabling more efficient use of constrained resources. We call for urgent, coordinated action to address challenges at the global, national, and procurement levels with tailored strategies that strengthen and expand NSPs across all settings. Scaling up NSPs is a public health necessity, a moral imperative, and a human rights obligation. Every person who injects drugs deserves access to sterile injecting equipment to protect their health and the health of their communities. The evidence is here. The tools are available. The time to act is now.

### Contributors

### Data sharing

The results of the barrier prioritisation survey are available upon request to the corresponding author.

## Declaration of interests

JG is supported by a National Health and Medical Research Council Investigator Grant (number 2034002); reports receiving grants or contracts from AbbVie, bioLytical, Cepheid, Gilead, and Hologic; and reports honoraria for lectures, presentations, or events from AbbVie, Abbott, Cepheid, Gilead, and Roche, all unrelated to this work. GF is supported by a Fonds de recherche du Québec – Santé Junior 1 Research Scholar Award. GF reports receiving hospitality from Gilead and speaker fees from AbbVie, unrelated to this work. MS reports receiving research grants or contracts from AbbVie and Gilead and consulting fees from Gilead, all unrelated to this work. PV reports receiving research grants and contracts from Gilead, unrelated to this work. NT reports receiving consulting fees from Gilead, unrelated to this work. AS reports receiving speaker fees from Gilead, unrelated to this work. ED, EW, KS, AM, and NL declare no competing interests.

## References

[1] Degenhardt L, Webb P, Colledge-Frisby S (2023). Epidemiology of injecting drug use, prevalence of injecting-related harm, and exposure to behavioural and environmental risks among people who inject drugs: a systematic review. Lancet Glob Health.

[2] Artenie A, Trickey A, Looker KJ (2025). Global, regional, and national estimates of hepatitis C virus (HCV) infection incidence among people who inject drugs and number of new annual HCV infections attributable to injecting drug use: a multi-stage analysis. Lancet Gastroenterol Hepatol.

[3] Artenie A, Stone J, Fraser H, the HIV and HCV Incidence Review Collaborative Group (2023). Incidence of HIV and hepatitis C virus among people who inject drugs, and associations with age and sex or gender: a global systematic review and meta-analysis. Lancet Gastroenterol Hepatol.

[4] Degenhardt L, Charlson F, Stanaway J (2016). Estimating the burden of disease attributable to injecting drug use as a risk factor for HIV, hepatitis C, and hepatitis B: findings from the Global Burden of Disease Study 2013. Lancet Infect Dis.

[5] Reddon H, Marshall BDL, Milloy MJ (2019). Elimination of HIV transmission through novel and established prevention strategies among people who inject drugs. Lancet HIV.

[6] Platt L, Minozzi S, Reed J (2018). Needle and syringe programmes and opioid substitution therapy for preventing HCV transmission among people who inject drugs: findings from a Cochrane review and meta-analysis. Addiction.

[7] Palmateer N, Hamill V, Bergenstrom A (2022). Interventions to prevent HIV and hepatitis C among people who inject drugs: latest evidence of effectiveness from a systematic review (2011 to 2020). Int J Drug Policy.

[8] Walker JG, Akiyama MJ, Artenie A (2025). Impact of scaling up harm reduction interventions on injecting risk behaviours, ART outcomes and HIV incidence among people who inject drugs in Kenya. Int J Drug Policy.

[9] McNaughton AL, Stone J, Oo KT (2023). Trends in HIV incidence following scale-up of harm reduction interventions among people who inject drugs in Kachin, Myanmar, 2008-2020: analysis of a retrospective cohort dataset. Lancet Reg Health West Pac.

[10] Colledge-Frisby S, Ottaviano S, Webb P (2023). Global coverage of interventions to prevent and manage drug-related harms among people who inject drugs: a systematic review. Lancet Glob Health.

[11] European Centre for Disease Prevention and Control, European Monitoring Centre for Drugs and Drug Addiction (November, 2023). Prevention and control of infectious diseases among people who inject drugs: 2023 update. https://www.ecdc.europa.eu/en/publications-data/prevention-and-control-infectious-diseases-among-people-who-inject-drugs-2023.

[12] Csete J, Kamarulzaman A, Kazatchkine M (2016). Public health and international drug policy. Lancet.

[13] Global Commission on Drug Policy (2023). HIV, hepatitis and drug policy reform. https://globalcommissionondrugs.org/gcdp-reports/hiv-hepatitis-drug-policy-reform/.

[14] Akiba CF, Smith J, Wenger LD (2024). Financial barriers, facilitators, and strategies among syringe services programs in the U.S., and their impact on implementation and health outcomes. SSM Qual Res Health.

[15] Baker P, Beletsky L, Avalos L (2020). Policing practices and risk of HIV infection among people who inject drugs. Epidemiol Rev.

[16] DeBeck K, Cheng T, Montaner JS (2017). HIV and the criminalisation of drug use among people who inject drugs: a systematic review. Lancet HIV.

[17] Jones CM (2019). Syringe services programs: an examination of legal, policy, and funding barriers in the midst of the evolving opioid crisis in the U.S. Int J Drug Policy.

[18] Yazdi-Feyzabadi V, Haghdoost A-A, McKee M (2025). The United States withdrawal from the World Health Organization: implications and challenges. Int J Health Policy Manag.

[19] International Network of People who Use Drugs (April 7, 2025). The human cost of policy shifts: the fallout of United States' foreign aid cuts on harm reduction programming and people who use drugs. https://idpc.net/publications/2025/04/the-human-cost-of-policy-shifts-the-fallout-of-us-foreign-aid-cuts-on-harm-reduction-programming.

[20] Buse K, Gostin L, Kamarulzaman A, McKee M (2025). The US withdrawal from the WHO: a global health crisis in the making. BMJ.

[21] Kickbusch I (2025). US exit from WHO: it is about much more than WHO. Lancet.

[22] Brink DT, Martin-Hughes R, Bowring AL (2025). Impact of an international HIV funding crisis on HIV infections and mortality in low-income and middle-income countries: a modelling study. Lancet HIV.

[23] Harm Reduction International (2023). The global state of harm reduction: 2023 update to key data. https://hri.global/publications/global-state-of-harm-reduction-2023-update-to-key-data/.

[24] Canadian Network on Hepatitis C (Sept 9, 2024). CanHepC statement on the Ontario Government's decision to ban supervised consumption sites. https://www.canhepc.ca/en/news/canhepc-statement-on-the-ontario-governments-decision-to-ban-supervised-consumption-sites.

[25] Hicks S, Abuna F, Odhiambo B (2023). Comparison of methods to engage diverse stakeholder populations in prioritizing PrEP implementation strategies for testing in resource-limited settings: a cross-sectional study. Implement Sci Commun.

[26] Gimbel S, Ásbjörnsdóttir K, Banek K (2023). The systems analysis and improvement approach: specifying core components of an implementation strategy to optimize care cascades in public health. Implement Sci Commun.

[27] Pokorny LJ, Lyle K, Tyler M, Topolski J (1988). Introducing a modified nominal group technique for issue identification. Eval Pract.

[28] Weerasuriya R, Elias J, Martyn M (2025). Facilitating equitable access to genomic testing for advanced cancer: a combined intuition and theory-informed approach to intervention development and deployment. Public Health Genomics.

[29] Mazariego C, Li Z, Ramanathan M, Johnston DA, Taylor N (2024). Optimising newborn screening consent in Queensland: results from two national workshops. University of New South Wales. https://www.unsw.edu.au/content/dam/pdfs/medicine-health/population-health/research-reports/2024-10-sph/Final-Consent-Workshops-Report%20.pdf.

[30] Fontaine G, Taylor N, Bruneau J (2025). The urgent need for implementation science to achieve hepatitis C elimination. Lancet Gastroenterol Hepatol.

[31] Fontaine G, Vinette B, Weight C (2024). Effects of implementation strategies on nursing practice and patient outcomes: a comprehensive systematic review and meta-analysis. Implement Sci.

[32] Chriqui JF, Asada Y, Smith NR, Kroll-Desrosiers A, Lemon SC (2023). Advancing the science of policy implementation: a call to action for the implementation science field. Transl Behav Med.

[33] Fontaine G, Mooney M, Porat-Dahlerbruch J (2025). Advancing the selection of implementation science theories, models, and frameworks: a scoping review and the development of the SELECT-IT meta-framework. Implement Sci.

[34] Damschroder LJ, Reardon CM, Widerquist MAO, Lowery J (2022). The updated Consolidated Framework for Implementation Research based on user feedback. Implement Sci.

[35] Damschroder LJ, Aron DC, Keith RE (2009). Fostering implementation of health services research findings into practice: a consolidated framework for advancing implementation science. ImplementSci.

[36] Powell BJ, Waltz TJ, Chinman MJ (2015). A refined compilation of implementation strategies: results from the Expert Recommendations for Implementing Change (ERIC) project. Implement Sci.

[37] Yakovchenko V, Lamorte C, Chinman MJ (2023). Comparing the CFIR-ERIC matching tool recommendations to real-world strategy effectiveness data: a mixed-methods study in the Veterans Health Administration. Implement Sci.

[38] Proctor EK, Powell BJ, McMillen JC (2013). Implementation strategies: recommendations for specifying and reporting. Implement Sci.

[39] Commission on Narcotic Drugs (2024). https://www.unodc.org/documents/commissions/CND/Drug_Resolutions/2020-2029/2024/Res_67_4.pdf.

[40] UNAIDS (2024). HIV and people who inject drugs. https://www.unaids.org/sites/default/files/media_asset/2024-unaids-global-aids-update-people-who-inject-drugs_en.pdf.

[41] Kerr P, Cossar RD, Livingston M, Jacka D, Dietze P, O'Keefe D (2022). Analysis of four syringe dispensing machine point-of-access data 2017–2020 in Melbourne, Australia: machine utilisation and client demographics. Harm Reduct J.

[42] Evans DS, Keenan E (February, 2024). https://www.drugsandalcohol.ie/40586/.

[43] WHO (2022). Global health sector strategies on, respectively, HIV, viral hepatitis and sexually transmitted infections, 2022–2030. https://www.who.int/teams/global-hiv-hepatitis-and-stis-programmes/strategies/global-health-sector-strategies.

[44] WHO, UN Office on Drugs and Crime, UNAIDS (Jan 2, 2012). WHO, UNODC, UNAIDS technical guide for countries to set targets for universal access to HIV prevention, treatment and care for injecting drug users – 2012 revision. https://www.who.int/publications/i/item/978924150437.

[45] Canada's Source for HIV and Hepatitis C Information (2023). CATIE statement on the distribution of a full range of harm reduction supplies as an evidence-based approach to reducing a range of preventable harms. https://www.catie.ca/catie-statement-on-the-distribution-of-a-full-range-of-harm-reduction-supplies-as-an-evidence-based.

[46] Strike C, Miskovic M, Perri M (2021). Best practice recommendations for Canadian programs that provide harm reduction supplies to people who use drugs and are at risk for HIV, HCV and other harms. Working Group on Best Practice for Harm Reduction Programs in Canada. https://www.catie.ca/best-practice-recommendations-for-canadian-harm-reduction-programs.

[47] National Institute for Health and Care Excellence (March 26, 2014). Needle and syringe programmes: public health guideline. https://www.nice.org.uk/guidance/ph52.

[48] Unitaid (2025). https://unitaid.org/project/catalyse-the-uptake-of-underutilised-tools-and-simplify-treatment-for-hepatitis-c/.

[49] Unitaid (2025). https://unitaid.org/project/preventing-hepatitis-c-through-harm-reduction/.

[50] Unitaid (2025). https://unitaid.org/project/hepatitis-c-combination-prevention-in-people-who-inject-drugs-and-incarcerated-populations/.

[51] Miskovic M, Zurba N, Beaumont D, Conway J (2020). https://ohrdp.ca/wp-content/uploads/2023/01/OHR-Connecting-FullGuide-English-WEB.pdf.

