# Supplementary material for: Addressing policy barriers to scaling up needle and syringe programmes: a global call to action
Source: Lancet Glob Health. 2025 Dec 9;14(1):e164–72. doi: 10.1016/S2214-109X(25)00433-4 (PMC12696260; doi:10.1016/S2214-109X(25)00433-4)
Supplement: Supplementary appendix [file mmc1.pdf]

THE LANCET
Global Health

Supplementary appendix

This appendix formed part of the original submission and has been peer reviewed. We post it as supplied by the authors.

Supplement to: Fontaine G, Day E, Luhmann N, et al. Addressing policy barriers to scaling up needle and syringe programmes: a global call to action. *Lancet Glob Health* 2026; **14:** e164–72.

Supplementary appendix

Table of contents

1. INHSU Policy Day Working Group Members 2

2. Characteristics of participants in the INHSU Policy Day in-person workshop......................... 3

3. Overview of the implementation science-informed methodology... 4

4. Full list of barriers to needle and syringe programme (NSP) access and engagement organized by domain.. 5

5. Full table of strategies, lead actors, outputs and outcomes... 6

6. Mapping of prioritized barriers to the Consolidated Framework for Implementation Research (CFIR) and the Expert Recommendations for Implementing Change Taxonomy (ERIC)............ 9

1. INHSU Policy Day Working Group Members

This appendix presents the full list of members of the International Network on Health and Hepatitis in Substance Users (INHSU) Policy Day Working Group who contributed to identifying and prioritizing barriers to scaling up needle and syringe programmes (NSPs) globally. One-on-one consultations were conducted with representatives engaged in the working group and present at the Policy Day. The International Network of People who Use Drugs (INPUD) was the primary community-based organisation consulted in this process. Other civil society, community-led organisations participated in the policy day workshop and reviewed post-policy day findings but did not participate in one-on-one consultations.

Table S1. Members of the INHSU Policy Day Working Group.

**Name**	**Primary affiliation(s)**	**Institutional base / country**	**Attended in person Policy Day**
Philip Bruggmann	ARUD (Swiss Association for Harm Reduction)	Switzerland	Yes
Judy Chang	International Network of People Who Use Drugs (INPUD)	Italy*	No
Emily Christie	Joint United Nations Programme on HIV/AIDS (UNAIDS)	Switzerland	No
Monica Ciupagea	United Nations Office on Drugs and Crime (UNODC)	Austria	Yes
Colleen Daniels	Harm Reduction International (HRI)	United Kingdom	Yes
Emma Day	International Network on Health and Hepatitis in Substance Users (INHSU)	Australia	Yes
Jason Grebely	INHSU / Kirby Institute, University of New South Wales	Australia	Yes
Kim Green	PATH	United States	No
Kiera Gustafson	PATH	United States	Yes
Jennifer Hasselgard-Rowe	Global Commission on Drug Policy	Switzerland	No
Niklas Luhmann	World Health Organization (WHO)	Switzerland	Yes
Annie Madden	INPUD	Australia*	Yes
Natasha Martin	University of California, San Diego	United States	Yes
Susie McLean	The Global Fund to Fight AIDS, Tuberculosis and Malaria	Switzerland	Yes
Keith Sabin	UNAIDS	Switzerland	No
Andrew Scheibe	INHSU / TB HIV Care	South Africa	Yes
Heather Marie Schmidt	UNAIDS	Switzerland	No
Mat Southwell	COACT, peer-led, technical-support agency working globally to strengthen harm-reduction responses for and with people who use drugs	United Kingdom	Yes
Mark Stoové	Burnet Institute	Australia	Yes
Karin Timmermans	Unitaid	Switzerland	Yes
Anna Tomasi	Global Commission on Drug Policy	Switzerland	No
Beatrix Vas	UNITE – Global Parliamentarians Network to End HIV/AIDS, TB and Malaria	Portugal	No
Annette Verster	WHO	Switzerland	Yes
Peter Vickerman	University of Bristol	United Kingdom	Yes
Ancella Voets	Mainline	Netherlands	No
Ernst Wisse	Médecins du Monde (Doctors of the World)	France	Yes

* INPUD is an international network headquartered in the UK, but both Judy Chang and Annie Madden are based in Italy and Australia respectively; the country column reflects their primary base of work.

2. Characteristics of participants in the INHSU Policy Day in-person workshop

The INHSU Policy Day in-person workshop was held in Athens, Greece on October 7, 2024, and included 54 participants.

Table S2. Characteristics of participants in the INHSU Policy Day in-person workshop.

Characteristic	Category	n	%
**World Bank income group**[1]	High income	32	59
	Low-/middle-income	22	41
**WHO region**	EURO (Europe & Central Asia)	22	41
	AFRO (Africa)	12	23
	WPRO (Western Pacific)	11	20
	EMRO (Eastern Mediterranean)	4	7
	AMRO (Americas)	4	7
	SEARO (South-East Asia)	1	2
**Type of organization**[2]	NGO / Community / Technical support	28	52
	Academic / Research	13	24
	Multilateral / UN / Global donor	6	11
	Government / Public-sector agency	5	9
	Other (e.g., hybrid or private entities)	2	4
**Primary role**	Community / Peer-led network representative	15	28
	Programme / Technical adviser	14	26
	Researcher / Academic faculty	13	24
	Multilateral / Donor staff	6	11
	Government official	5	9
	Other	1	2

[1] Based on World Bank 2025 income classifications using each participant’s primary country of work.
[2] Organizations were categorized according to their main function. “NGO / Community / Technical support” includes peer-led networks, international NGOs, and technical support agencies such as COACT and PATH.

3. Overview of the implementation science-informed methodology

The figure presents the implementation science-informed methodology used to identify and prioritize barriers to NSP scale-up, and to co-design context-sensitive strategies for enhancing coverage globally.

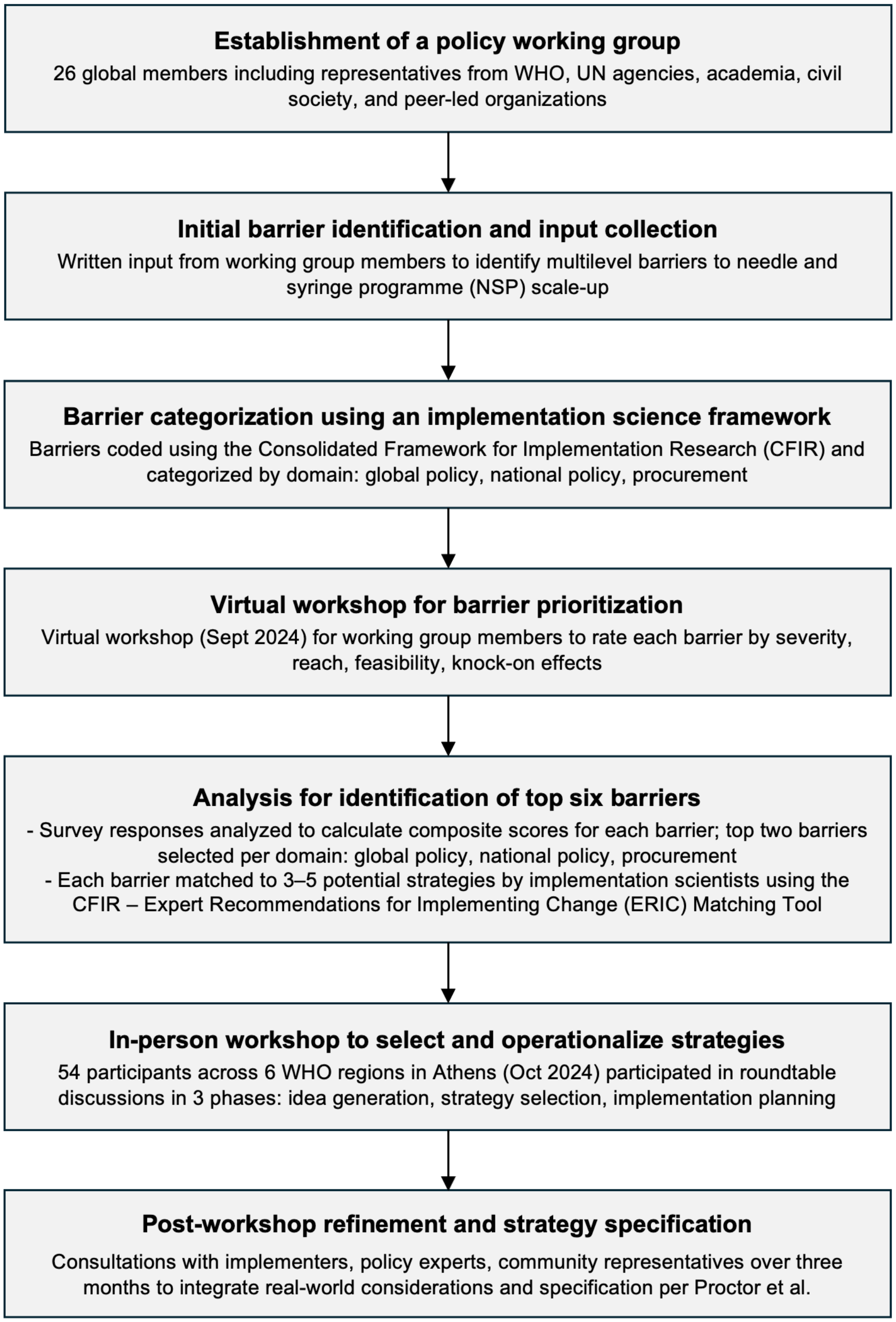

Figure S1. Overview of the implementation-science informed methodology.

4. Full list of barriers to needle and syringe programme (NSP) access and engagement organized by domain

Table S3. Key and prioritised barriers to NSP access and engagement organized by domain and focus areas generated by the working group.

Key focus areas	Barriers	Composite score (0–5)<br>Severity: 30%<br>Reach: 10%<br>Positive Effects: 10%<br>Feasibility: 50%	Prioritized for strategy development
Global Policy Barriers	***Absence of global guidance for selecting and procuring NSP commodities preferred by people who inject drugs***	***3.18***	***Yes***
	Restrictive global funder policies that prevent allocation of resources to NSPs and harm reduction (e.g., federal constraints on PEPFAR funding for NSPs)	***3.09***	***Yes***
	Global drug policy focus on the criminalization of drug use	2.91	No
	Limited real-world evidence on the effectiveness of low-dead-space (LDS) syringes in reducing blood-borne virus (BBV) risk among people who inject drugs to inform policy	2.52	No
	Historical purchasing demand (i.e. lack of demand for varied commodities) impacts global manufacturing and supply chain	2.36	No
National Policy Barriers	***Lack of political will from national governments to allocate or request resources for NSP implementation or scale-up***	***3.09***	***Yes***
	Restrictive policies or practices limiting NSP access such as 1:1 exchange or identification mandates	***3.03***	***Yes***
	Lack of commitment to supporting varied NSP services and distribution avenues including standalone services, pharmacy NSPs, vending machines, peer outreach, mobile services, secondary distribution due to lack of understanding, lack of will, or cost of various models	2.79	No
	Opposition to harm reduction agenda from interest-holders such as citizens and police	2.62	No
	Lack of knowledge about different types of needles and syringes at the national policy level	2.59	No
	Restrictive monitoring and reporting mandates preventing tailored responsive service delivery	2.16	No
Procurement Barriers	***Insufficient commodity quantities requested by country recipients from global funding organisations to meet WHO coverage goals***	***3.25***	***Yes***
	Country-level procurement processes that do not prioritise community-acceptable and appropriate equipment	***2.99***	***Yes***
	Lack of understanding about available modelling to inform country requests that are based on local data and demand	2.44	No
	Limited understanding of the impact of global supply chain and regional manufacturing (if feasible) on the price and availability of quality assured low dead space syringes and needles in low and middle-income countries	2.21	No

5. Full table of strategies, lead actors, outputs and outcomes

Legend — Lead actor symbols

- ● Community networks of people who use drugs (e.g., International of People who Use Drugs [INPUD])
- □ World Health Organization (WHO)
- ▲ The Global Fund and other health funds
- ☆ Harm reduction advocacy groups (e.g., Harm Reduction International [HRI])
- + Clinical and research networks (e.g., International Network on Health and Hepatitis in Substance Users [INHSU])
- ⬡ Technical nonprofit organizations (e.g., PATH)
- ◆ Country coalitions and government partners

Table S4. Strategies for NSP scale-up aligned with the six prioritized global policy, national policy and procurement barriers.[1]

Barriers	Strategies (S1–S11)	Lead Actors	Outputs	Outcomes
Global policy – Absence of client-centred guidelines from global funders, governments, and other interest-holders for selecting and procuring NSP commodities preferred by people who inject drugs	S1. Engage people who inject drugs in developing NSP commodity selection procurement guidelines to align with community needs and preferences.	● □	• Revised commodity selection and procurement guidelines prioritizing community-centred criteria.	• Increased relevance and acceptability of commodities selected for distribution in NSPs.<br>• Increased procurement accuracy, reducing costs and wastage.<br>• Increased uptake of NSP services and use of sterile injecting equipment by people who use drugs.
	S2. Refine the Global Fund NSP funding request process by requiring donor recipients to justify equipment type and quantity based on community values, preferences, and data-driven demand estimates.	● ▲	• Revised Global Fund application framework incorporating community input in NSP commodity requests.	• Improved alignment of NSP commodity requests with community needs and preferences, increasing uptake.<br>• Improved accuracy of NSP demand estimates, optimizing funding allocations.<br>• Enhanced capacity of country-level recipients to engage people who inject drugs in procurement planning.
Global policy – Restrictive global funder policies that prevent allocation of resources to NSPs and harm reduction	S3. Engage global funding organizations to revise policies, ensuring they adopt the UN Commission on Narcotic Drugs' harm reduction resolution, recognizing harm reduction as a distinct funding priority.	● ☆	• Coalition of international interest-holders advocating for global funding policy change.	• Adoption of harm reduction as a standalone funding priority by key global funders.<br>• Increased availability of dedicated funding for harm reduction services, separate from HIV program budgets.<br>• More stable and sustainable funding channels, enhancing long-term program effectiveness.

	S4. Develop knowledge-strengthening programs targeting global funders to increase their understanding of harm reduction, its evidence-based benefits, and alignment with public health goals.	● ☆ +	• Harm reduction knowledge-strengthening program and delivery strategy suitable to global funders.	• Greater awareness and support for harm reduction among global funders.<br>• Policy shifts allowing increased or dedicated funding for NSPs.
National policy – Lack of political will from national governments to allocate or request resources for NSP implementation or scale-up	S5. Advocate through a coordinated, multi-interest-holder effort to secure government allocation or requests for national NSP funding.	◆ ● ☆ +	• Country-level strategic advocacy plan that aligns coalition members around common goals and consistent messaging.	• A coalition bringing together expertise and influence from various sectors.<br>• A cohesive advocacy approach with clear, unified messaging.<br>• Increased visibility for NSPs through coordinated lobbying and direct engagement with policymakers.
	S6. Facilitate consensus-building discussions between national governments, healthcare providers, international organizations, and community interest-holders to create a shared understanding of the impact of NSPs and their investment case.	◆ ● ☆ +	• A consensus statement that outlines commitments, actions, and timelines for NSP scale-up.	• Strengthened political commitment to NSP funding.<br>• Cross-sectoral alignment on NSP goals, with engagement from governments, healthcare providers, international organizations, and community.<br>• Clear resource allocation priorities for harm reduction.<br>• More sustainable funding and efficient allocation of NSP resources.
National policy – Restrictive policies or practices limiting NSP access such as 1:1 exchange or identification mandates	S7. Collaborate with governments, funders, and policymakers to develop national guidelines that encourage the adoption of less restrictive policies for NSP access (e.g., peer delivery, dispensing machines, coupon distribution, simplify process for organisations to register as an NSP service).	◆ ● ☆ +	• Updated national guidelines promoting less restrictive NSP practices.	• Increased adoption of community-led NSP models.<br>• Enhanced accessibility to NSP services, reducing barriers for people who inject drugs.<br>• Strengthened collaboration among governments, funders, and harm reduction advocates for evidence-based policy reform.
Procurement – Insufficient commodity quantities requested by country recipients from	S8. Develop a quantification tool to support accurate NSP commodity requests, ensuring alignment with actual usage patterns.	□	• NSP quantification tool to be used in WHO NSP Operational Guide and Global Fund application process.	• Improved accuracy in NSP commodity forecasting and allocation.<br>• Better alignment of funding requests with actual service needs, reducing shortages and waste.

global funding organisations to meet WHO coverage goals				• Strengthened advocacy for NSP funding through data-driven resource planning.<br>• Standardized practices in NSP commodity allocation.<br>• Use of quantification tool for investment case.
	S9. Implement targeted training sessions on using the WHO NSP Operational Guide, quantification tool, strategic commodity planning, including engagement with local communities to inform demand.	□ ▲ ● +	• Training program for national interest-holders requesting NSP funding.	• Enhanced capacity of national health and procurement officers in commodity forecasting and procurement.<br>• More efficient and strategic NSP commodity planning at the national level.<br>• Increased alignment with WHO guidelines and Global Fund requirements.<br>• Strengthened NSP implementation, reducing gaps and improving delivery.
Procurement – Country-level procurement processes that do not prioritise community-acceptable and appropriate equipment, undermining uptake and high NSP coverage	S10. Engage NSP suppliers and global procurers to establish optimal quality standards, policies, and benchmark pricing for preferred products.	⬡ ●	• Recommended NSP commodity and price list, with guidance on aligning commodities to drug use practices.<br>• Business case for manufacturers and suppliers to prioritize PWID-preferred products.<br>• Market-shaping interventions to increase manufacturer commitment for comparably priced, quality assured, and community-informed products to LMICs.	• Improved quality and affordability of NSP commodities globally.<br>• Increased manufacturer alignment with harm reduction priorities.<br>• Enhanced access to a range of high-quality NSP products.<br>• Strengthened market sustainability for local production.
	S11. Develop and implement a community-led values and preferences tool with funded technical assistance to enable routine, low-burden client-preference data for commodity requests.	●	• Community-led values and preferences tool integrated into WHO NSP guidelines, Global Fund application guidelines, and country-level procurement processes.	• Greater alignment of commodity requests with community preferences.<br>• Enhanced community engagement in procurement processes.<br>• Improved acceptance and utilisation of NSP commodities.<br>• Strengthened evidence base for NSP procurement.

1. Extract from the INHSU Policy Day Report.[42]

6. Mapping of prioritized barriers to the Consolidated Framework for Implementation Research (CFIR) and the Expert Recommendations for Implementing Change Taxonomy (ERIC)

For each prioritized barrier, we first mapped the barrier to one or more constructs from the original Consolidated Framework for Implementation Research (CFIR v1; Damschroder et al., 2009). We then used the CFIR–ERIC Matching Tool (Waltz et al., 2019), which links CFIR v1 constructs to 73 strategies in the Expert Recommendations for Implementing Change (ERIC) taxonomy based on endorsements from implementation science experts, to identify candidate strategies (Powell et al., 2015). For each construct, the tool provides a ranked list of ERIC strategies and the percentage of experts who endorsed each strategy as appropriate for addressing that construct (0–100%), based on the study by Waltz et al. (2019). Rather than applying a fixed percentage threshold, we selected five strategies the highest-ranked strategies for each construct (typically within the top 10 to 15 recommendations), because some constructs had relatively low maximum endorsement (e.g., up to 41%), consistent with the diversity in expert recommendations reported by Waltz and colleagues. We then reviewed this candidate set with the project team for conceptual fit with NSP scale-up and refined wording. These potential strategies were distributed to each table during the in-person workshop, and participants could select and further refine strategies from this set based on their expertise and local context.

- **Expert Recommendations for Implementing Change (ERIC) Taxonomy:** Powell, B.J., Waltz, T.J., Chinman, M.J. *et al.* A refined compilation of implementation strategies: results from the Expert Recommendations for Implementing Change (ERIC) project. *Implementation Sci* **10**, 21 (2015). https://doi.org/10.1186/s13012-015-0209-1
- **Original Consolidated Framework for Implementation Research (CFIR v1):** Damschroder, L.J., Aron, D.C., Keith, R.E. *et al.* Fostering implementation of health services research findings into practice: a consolidated framework for advancing implementation science. *Implementation Sci* **4**, 50 (2009). https://doi.org/10.1186/1748-5908-4-50
- **CFIR – ERIC Matching Tool:** Waltz, T.J., Powell, B.J., Fernández, M.E. *et al.* Choosing implementation strategies to address contextual barriers: diversity in recommendations and future directions. *Implementation Sci* 14, 42 (2019). https://doi.org/10.1186/s13012-019-0892-4

Table S5. Mapping to prioritized barriers to the domains and constructs of the Consolidated Framework for Implementation Research (CFIR) and potential strategies in the Expert Recommendations for Implementing Change (ERIC) taxonomy.

Barrier	CFIR Domain(s)	CFIR Construct(s)	Potential ERIC strategies to address barrier to be brought forward for discussion and refinement during the in-person workshop
Global policy – Absence of client-centred guidelines from global funders, governments, and other interest-holders for selecting and procuring needle and syringe programme (NSP) commodities preferred by people who inject drugs	Outer Setting	External Policy & Incentives, Peer Pressure, Patient Needs & Resources	• **Build a coalition** *(CFIR–ERIC: 33%[1]; 16%[2]; 14%[3])*: Bring together global stakeholders, including funders, policymakers, suppliers, healthcare providers, and harm reduction advocates, to develop guidelines and systems.<br>• **Involve executive boards** *(CFIR–ERIC: 41%[1]; 28%[2]; 5%[3])*: Engage the executive boards of relevant funding organizations to ensure strategic alignment and prioritization for developing and implementing NSP commodity guidelines.<br>• **Involve patients/consumers** *(CFIR–ERIC: 71%[3]; 28%[2]; 11%[1])*: Include people who inject drugs and harm reduction advocacy groups in guideline development.<br>• **Model and simulate change** *(CFIR–ERIC: 13%[2]; 4%[1])*: Use system dynamics modelling and simulation exercises to explore the potential impacts of new NSP commodity procurement systems and policies.<br>• **Obtain formal commitments** *(CFIR–ERIC: 15%[1]; 3%[2])*: Secure commitments from global stakeholders, including

			suppliers, manufacturers, and funders, to ensure consistent availability and transparent procurement processes.
Global policy – Restrictive global funder policies that prevent allocation of resources to NSPs and harm reduction	Outer Setting	External Policy & Incentives	• **Build a coalition** *(CFIR–ERIC: 33%[1])*: Bring together global stakeholders, including funders, policymakers, suppliers, healthcare providers, and harm reduction advocates, to improve NSP commodity procurement systems.<br>• **Capture and share local knowledge** *(CFIR–ERIC: 26%[1])*: Carry out cost–benefit and impact analyses to quantify the public health and economic benefits of revising restrictive NSP policies and use these data to advocate for policy change at global and national levels.<br>• **Involve executive boards** *(CFIR–ERIC: 41%[1])*: Engage executive boards to review and revise restrictive funding policies.<br>• **Obtain formal commitments** *(CFIR–ERIC: 15%[1])*: Secure written and public commitments to modify or eliminate policies that prevent equitable resource distribution for NSPs and harm reduction services.<br>• **Use advisory boards and workgroups** *(CFIR–ERIC: 15%[1])*: Establish a global taskforce to develop standardized technical specifications, quantification methodologies, and procurement guidelines tailored to NSP commodities.
National policy – Lack of political will from national governments to allocate or request resources for NSP implementation or scale-up	Outer Setting	External Policy & Incentives, Peer Pressure	• **Build a coalition** *(CFIR–ERIC: 33%[1]; 16%[2])*: Form a coalition of governments, NGOs, healthcare providers, and harm reduction experts to advocate for political commitment and resource allocation<br>• **Conduct local consensus discussions** *(CFIR–ERIC: 28%[2]; 22%[1])*: Facilitate discussions between governments, providers, and communities to agree on priorities for harm reduction services<br>• **Identify and prepare champions** *(CFIR–ERIC: 38%[2]; 22%[1])*: Identify and support influential leaders to advocate for policy reform and resource allocation using evidence and advocacy tools)<br>• **Obtain formal commitments** *(CFIR–ERIC: 15%[1], 3%[2])*: Secure written commitments from governments and organizations to allocate resources and support harm reduction policies)<br>• **Use mass media** *(CFIR–ERIC: 15%[1]; 13%[2])*: Launch a media campaign with evidence-based messages and local testimonials to raise awareness and build political support)
National policy – Restrictive policies or practices limiting NSP access such as 1:1 exchange or identification mandates	Outer Setting	External Policy & Incentives	• **Alter incentive/allowance structures** *(CFIR–ERIC: 41%[1])*: Work with policymakers to remove restrictive policies (e.g., 1:1 exchange) by creating financial incentives for more accessible NSP models<br>• **Conduct local consensus discussions** *(CFIR–ERIC: 7%[1])*: Organize discussions among stakeholders to address the impacts of restrictive policies and build support for reform<br>• **Develop disincentives** *(CFIR–ERIC: 7%[1])*: Tie funding to the removal of restrictive NSP policies to encourage the adoption of best practices<br>• **Involve patients/consumers** *(CFIR–ERIC: 11%[1])*: Engage people who inject drugs, peers, and advocacy

			groups in policymaking through community boards and consultations<br>• **Stage implementation scale-up** *(CFIR–ERIC: 4%[1])*: Pilot the removal of restrictive NSP policies at select sites and evaluate impacts to support broader changes
Procurement – Insufficient commodity quantities requested by country recipients from global funding organisations to meet WHO coverage goals	Outer Setting, Inner Setting, Process	External Policy & Incentives, Networks & Communications, Reflecting & Evaluating	• **Alter incentive/allowance structures** *(CFIR–ERIC: 41%[1])*: Develop incentives like additional funding or technical support for countries that align their requests with WHO targets<br>• **Centralize technical assistance** *(CFIR–ERIC: 26%[4])*: Set up a network of technical experts to support countries with procurement planning, quantification, and supply chain management<br>• **Conduct educational meetings** *(CFIR–ERIC: 15%[1]; 13%[4])*: Design training for health and procurement officers on forecasting and strategic planning to ensure accurate requests for health commodities<br>• **Conduct local consensus discussions** *(CFIR–ERIC: 22%[1,4])*: Involve local health leaders, government representatives, and international organizations in discussions to align on the need for proper commodity requests to meet coverage goals<br>• **Use data experts** *(CFIR–ERIC: 28%[5])*: Use real-time data and case studies to highlight gaps in commodity requests and their impact on health outcomes. Use these insights to advocate for better forecasting and target alignment.
Procurement – Country-level procurement processes that do not prioritise community-acceptable and appropriate equipment, undermining uptake and high NSP coverage	Outer Setting	Patient Needs & Resources, External Policy & Incentives	• **Capture and share local knowledge** *(CFIR–ERIC: 26%[1]; 10%[3])*: Collect and share insights from local healthcare providers and procurement officers on which products work best in specific settings to influence broader procurement practices<br>• **Conduct local needs assessments** *(CFIR–ERIC: 57%[3]; 7%[1])*: Perform detailed assessments at the community level to understand local preferences and specific requirements for equipment used in healthcare<br>• **Develop and implement tools for quality monitoring** *(CFIR–ERIC: 14%[3]; 11%[1])*: Create an evaluation framework for community members, providers, and procurement teams to evaluate the suitability of newly procured equipment and provide continuous feedback<br>• **Involve patients/consumers** *(CFIR–ERIC: 71%[3]; 11%[1])*: Actively include people who inject drugs and advocacy groups in defining product specifications to ensure the equipment meets local needs and cultural expectations<br>• **Obtain formal commitments** *(CFIR–ERIC: 15%[1])*: Secure commitments from national procurement agencies to prioritize culturally relevant specifications that meet community needs

1. For the CFIR construct “External Policy & Incentives.”
2. For the CFIR construct “Peer Pressure.”
3. For the CFIR construct “Patient Needs & Resources.”
4. For the CFIR construct “Networks & Communications.”
5. For the CFIR construct “Reflecting & Evaluating.”
